# Variation in Extrafloral Nectary Productivity Influences the Ant Foraging

**DOI:** 10.1371/journal.pone.0169492

**Published:** 2017-01-03

**Authors:** Denise Lange, Eduardo Soares Calixto, Kleber Del-Claro

**Affiliations:** 1 Universidade Tecnológica Federal do Paraná, Campus Santa Helena, Santa Helena, PR, Brazil; 2 Pós-Graduação em Entomologia, Faculdade de Filosofia, Ciências e Letras, Universidade de São Paulo, Ribeirão Preto, SP, Brazil; 3 Laboratório de Ecologia Comportamental e de Interações (LECI), Instituto de Biologia, Universidade Federal de Uberlândia, Uberlândia, MG, Brazil; Indian Institute of Science, INDIA

## Abstract

Extrafloral nectar is the main food source offered by plants to predatory ants in most land environments. Although many studies have demonstrated the importance of extrafloral nectaries (EFNs) to plant defense against herbivores, the influence of EFNs secretory activity pattern on predatory ants remains yet not fully understood. Here, we verified the relation between the extrafloral nectar production of a plant community in Cerrado in different times of the day, and its attractiveness to ants. The extrafloral nectaries (EFNs) of seven plant species showed higher productivity overnight. Ant abundance was higher in times of large extrafloral nectar production, however, there was no positive relation between ant richness on plants and EFNs productivity. There was temporal resource partitioning among ant species, and it indicates strong resource competition. The nectar productivity varied among plant species and time of the day, and it influenced the visitation patterns of ants. Therefore, EFNs are a key ant-plant interaction driver in the studied system.

## Introduction

Extrafloral nectar may be a key driver of insect-plant protective mutualisms [1–3]. This substance is produced at glandular structures called extrafloral nectaries (EFNs), which highly differ from each other in structure and morphology [4–6]. These glands may be found in all plant organs above the ground and their function is not related to pollination [7]. These nectaries are found in 3,941 species belonging to 108 different families of vascular plant species [8]. The EFN-bearing plants may represent 31% of the individuals and 25% of the arboreal plant species in tropical ecosystems, especially in Cerrado areas [9].

Extrafloral nectar is rich in carbohydrates, amino acids and lipids, among other organic compounds [10–11], and it is often consumed as food by a wide diversity of ants [3, 12–13], spiders [14–15] and other arthropods [16–18]. According to Blüthgen et al. [19], extrafloral nectar is probably the most common food available to ants in plants. It has been assumed that these nectaries help the ecological dominance of ants worldwide [20]. The visitation of EFNs-attracted ants may favor the host plants, because these insects prey on herbivores or reduce their activity on plants [21]. Indeed, ants are considered to be the major biotic defense against herbivory in EFN-bearing plants [21, 22–24], although spiders may also play the same role [14–15].

Although many studies have demonstrated the importance of EFNs-attracted ants to plant defense against herbivores [25–26], the influence of the EFN secretory activity pattern on ants remains yet not fully understood. According to O’Dowd [27], “if a primary function of foliar nectar production is the maintenance on the plant of potential anti-herbivore agents such as ants, then the deciphering of relationships among nectar production, ant visitation and leaf development is particularly important”. We must also take into consideration that the extrafloral nectar may be a major nutrient to the development and survival of arboreal ants [28].

Therefore, we assessed the nectar production at the EFNs of the most common and abundant trees in a tropical savanna (Cerrado) and its attractiveness to ant communities. We asked the following questions: (i) what is the extrafloral nectar productivity (volume and sugar concentration) of the main representative plants of this community? (ii) is there extrafloral nectar productivity variation throughout the day and among plant species? (iii) is there a link between extrafloral nectar productivity and ant visitation to plants? Our main hypothesis is that EFN productivity changes depending on the plant species and on the time of the day, and such variation influences the presence and richness of foraging ants on these plants.

## Materials and Methods

### Study area and plant species

The field work was conducted at Clube Caça e Pesca Itororó de Uberlândia (CCPIU) ecological reserve (18°57'45''S, 48°17'30''W), Uberlândia, Minas Gerais State, Brazil. The Biology Institute of Universidade Federal de Uberlândia has a memorandum of understanding with CCPIU, an agreement between board of directors of CCPIU, and Kleber Del Claro, director of Biology Institute that enables ecological studies in the area. The data was collected from September to November 2010 period of greatest EFN activity in most of the trees in the study site (see Lange *et al*. 2013), where Cerrado stricto sensu is the dominant vegetation. At this site, trees reach heights of 2–8 m, with an understory of shrubs and grasses [29]. The climate is marked by a rainy season, from September to March, and by a dry season, from April to August [30].

We selected the seven most abundant EFN-bearing tree species in the study site (see [29], namely: *Qualea multiflora* (Mart.) (Vochysiaceae), *Qualea grandiflora* (Mart.) (Vochysiaceae), *Ouratea spectabilis* (Mart.) Engl. (Ochnaceae), *Ouratea hexasperma* (A. St.-Hil.) Baill (Ochnaceae), *Stryphnodendron polyphyllum* (Mart.) (Fabaceae), *Stryphnodendron adstringens* (Mart.) Coville (Fabaceae) and *Lafoensia pacari* (A. St.-Hil.) (Lythraceae). The location of the EFNs in most of these species and their morphology were described by Machado et al. [9] and Oliveira and Leitão-Filho [5], respectively. Most of these species are deciduous and they lose their leaves between May and August, with releafing in September, at the beginning of the rainy season. *Ouratea spectabilis* and *O*. *hexasperma* are not deciduous, but they produce new leaves and active EFNs at the same period as the other species do [31].

### EFNs productivity and ant activity

We assessed three EFNs on lateral branches of ten individuals, from each of the seven selected species, which have presented similar architecture. Each of the assessed EFNs came from three distinct branches of each plant. The observation procedures took place when the selected individuals presented 30 to 50% of foliar resprouting. We followed the selected EFNs from the beginning of their activity until their necrosis (sensu [9]). We washed the gland in distilled water and bagged it after its activity had begun. Nectar production was measured three times a day: 6:00 am, 2:00 pm and 10:00 pm. We measured EFN production every two days until the nectaries no longer functioned. We did not collect nectar on a daily basis in order to prevent EFN production stimulus through constant removal, as it was mentioned by [32–33]. We measured nectar volume using calibrated microcapillary tubes; sugar concentration was measured using a refractometer (Eclipse®). We washed the EFNs in distilled water after each measurement and dried them on filter paper to ensure that the nectar production referred to the correct sample collection period. Then, we used the techniques developed by Büthgen et al. [10] and Bixenmann et al. [33], which isolated the branches in voile bags to prevent the removal of nectar for animals, as well as from being diluted by rain and dew.

We assessed EFN productivity, as well as the abundance and richness of ants on plants, on the same day. We collected one individual from each ant species and stored it in 70% alcohol for further identification. The ants collected not involve endangered or protected species. We also collected temperature and humidity data during the surveys.

### Data analysis

We used the nectar volume and the sugar concentration of each collected solution to calculate the sucrose equivalent, which corresponds to the number of sugar milligrams per microliter, as it was suggested by [34]. We performed the calculation according to the method described by [35] and used the following equation: *y* = 0.00226 + (0.00937 *x*) + (0.0000585 *x*^*2*^), where in “x” is the concentration value (refractometer reading) and “y” is the amount of total sugars in 1μL. Subsequently, we multiplied the results of the aforementioned calculation by the total volume of solution. Finally, the result of this last calculation was multiplied by four in order to find the solution energy value in calories, i.e., each 1 mg of sugar is equal to four calories (see [35]). We did not measure the solution concentration when nectaries showed very low volume (less than or equal to 0.1 μL), in such cases, we just used the volume in the analysis.

We used the two-way ANOVA to compare the total volume and the amount of calories produced at the EFNs of the seven plant species in the different time of day. We compared the total EFN productivity volume and the amount of calories in the assessed periods (10:00 pm– 6:00 am; 6:00 am– 02:00 pm; and 02:00 pm– 10:00 pm) through the Friedman test (F). We used the means of three EFNs from each plant in these analyses, and it corresponded to ten independent samples per plant species. The one-way ANOVA was used to compare the relative frequency of ant species among plant species and to compare the total volume and the amount of calories produced at the EFNs of the seven plant species. We did not use surveys that lack nectar in the nectary when we analyzed sugar concentration in the nectar and calories. We used Kruskal-Wallis test (H) to compare the number of surveys presenting active EFN among species. The Pearson’s correlation test was used to check the link among the total frequency of ants, the total volume of nectar and the total quantity of energy produced by each species (n = 7). We conducted all the analyses in the Systat 12.0 software, and 5% probability significance level was taken into account. The analyses were chosen after the assumptions of the parametric test where we used Lilliefors’ test (5%) for normality and Levene’ test for equality of variances.

## Results

### EFN productivity

**Fig 1:**
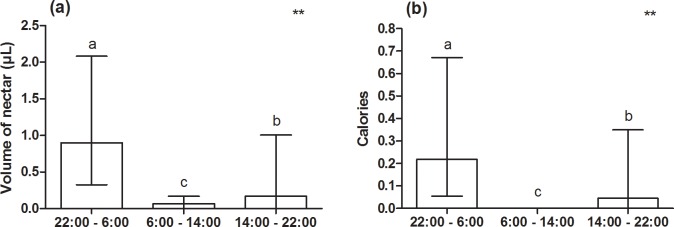
**Total volume of nectar (a) and quantity of calories (b) produced at the EFNs in the group of plants (seven species) assessed at three distinct times of the day.** The bars represent the median and the interquartile range. Friedman test F = 59.36; n = 70; p < 0.001 for (a), and F = 63.65; n = 64; p < 0.001. The letters represent the difference among the three times of the day according to Dunn’s post-hoc.

**Fig 2:**
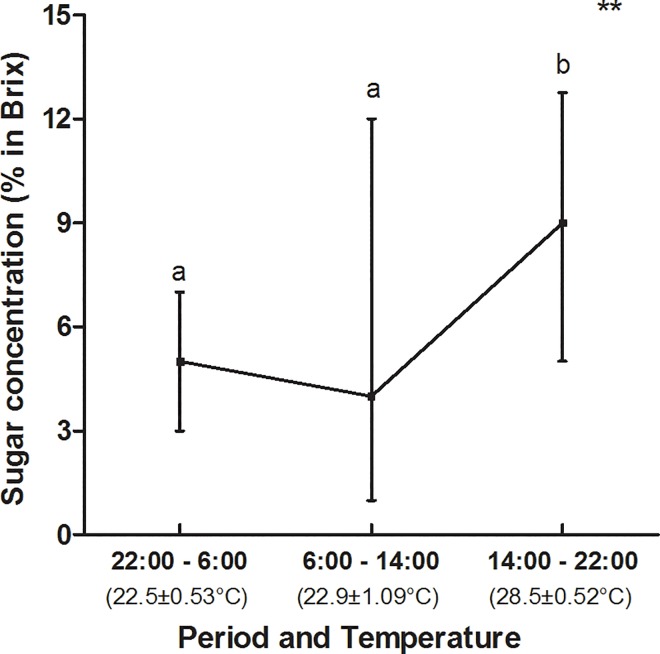
Sugar concentration (% in Brix) in the nectar produced at the EFNs in the group of plants (seven species) assessed at three distinct times of the day. The lines among periods connect the medians. The vertical line represents the interquartile range. Kruskal-Wallis test, H = 44.67; df = 2; p < 0.001 (**). The letters refer to differences between the treatment periods–those with the same letters are not significantly different according to Dunn’s post-hoc. The X-axis numbers in parentheses show the mean temperature (mean ± SE) in each period.

We recorded 232.35 Kcal in 573.45 μL of nectar collected in 210 EFNs from 70 individuals (10 individuals per species), according to the sum of all studied plant species (n = 7). The volume of nectar produced by this group of plants was different in each of the three assessed periods (Friedman test, F = 59.36; n = 70; p < 0.001 –Fig 1A) and the highest volumes were recorded between 10:00 pm and 6:00 am (1.50 ± 0.20 μL) and between 02:00 pm and 10:00 pm (1.02 ± 0.24 μL), and the lowest one between 6:00 am and 02:00 pm (0.20 ± 0.05 μL) (mean ± SE). Likewise, the amount of calories in the nectar was different depending on the time of the day (Friedman test, F = 63.65; n = 64; p < 0.001) (Fig 1B). Sugar concentration in the nectar was also different depending on the time of the day and it presented the highest values in the times of higher temperatures (Kruskal-Wallis test, H = 44.67; df = 2; p < 0.001) (Fig 2).

**Fig 3:**
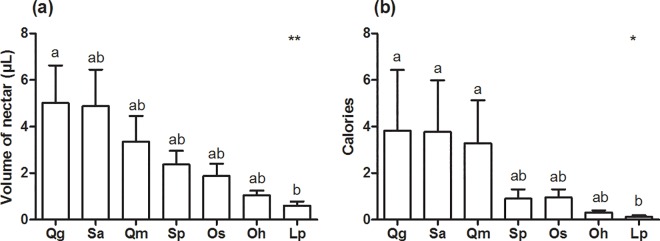
**The total volume of nectar (a) and quantity of calories (b) produced at the EFNs in the seven assessed plant species.** (*Qg*) *Qualea grandiflora*, (*Sa*) *Stryphnodendron adstringens*, (*Qm*) *Q*. *multiflora*. (*Sp*) *S*. *polyphyllum*, (*Os*) *Ouratea spectabilis*, (*Oh*) *O*. *hexasperma*, and (*Lp*) *Lafoensia pacari*. Data were collected from September to November 2010 in Uberlândia, Minas Gerais State, Brazil. The bars represent the mean and the SE. One-way ANOVA F_[__6__,_ _69__]_ = 3.03; p < 0.001 for (a, **), and F[_6__,_ _63__]_ = 4.33; p = 0.01 for (b, *). The letters represent the difference among the three periods according to Tukey’s post-hoc.

We found that the seven assessed species produced different nectar volumes but similar calorie quantities (see results of two-way ANOVA in Table 1). The plant species produced nectar (volume and calorie quantities) at different times of day. We found that the seven assessed species produced different total nectar volumes and calorie quantities: two-way ANOVA, F_[__6__,_ _69__]_ = 3.03, p < 0.001) (Fig 3A), for volume; and F_[__6__,_ _63__]_ = 4.33, p < 0.01 (Fig 3B), for calories. *Qualea grandiflora* and *S*. *adstringens* were the species presenting the highest nectar production (Fig 3A). The nectar produced by these two species had higher sugar concentration, and this resulted in greater caloric amounts (Fig 3B). On the other hand, *O*. *hexasperma* and *L*. *pacari* were the species that produced lower volumes and lesser calories.

**Table 1:** Results of two-way ANOVA used to compare the effects of species, time and both on volume of nectar and quantity of calories produced at the EFNs of seven species assessed at three distinct times of the day.

Source	df	SS	F	P-value
**Volume**				
Time	2	60.79	21.09	<0.0001
Species	6	61.21	3.034	0.0114
Time x Species	12	52.96	3.061	0.0008
Error	126	181.6		
**Calories**				
Time	2	31.38	4.538	0.012
Species	6	55.82	9.304	0.262
Time x Species	12	63.92	1.540	0.120
Error	112	387.3		

The majority of the EFNs remained active for approximately two days. However, some EFNs remained active for 16 consecutive days. We found no difference in the number of days each EFN remained active among species (Kruskal-Wallis test, H = 9.07; df = 6; p = 0.169): *S*. *adstringens* (2.03 ± 1.71 assessment days–for 16 activity days), *Q*. *multiflora* (1.84 ± 1.29, 16 days), *S*. *polyphyllum* (1.8 ± 1.62, 16 days), *O*. *hexasperma* (2.05 ± 1.22, 13 days), *Q*. *grandiflora* (1.96 ± 1.11, 13 days), *O*. *spectabilis* (1.92 ± 1.14, 13 days), and *L*. *pacari* (1.35 ± 0.67, 13 days) (mean ± SD).

**Fig 4:**
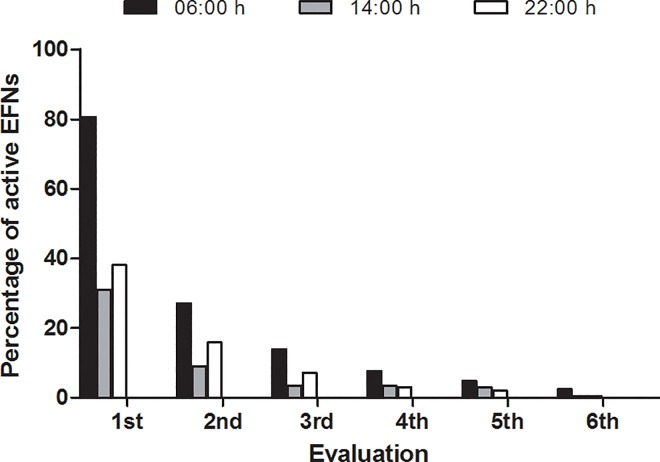
Percentage of active EFNs (n = 210 EFNs) in the group of plant species (n = 7 species) assessed at three distinct times of the day since the beginning of their activity up to their necrosis (n = 6 observations).

**Fig 5:**
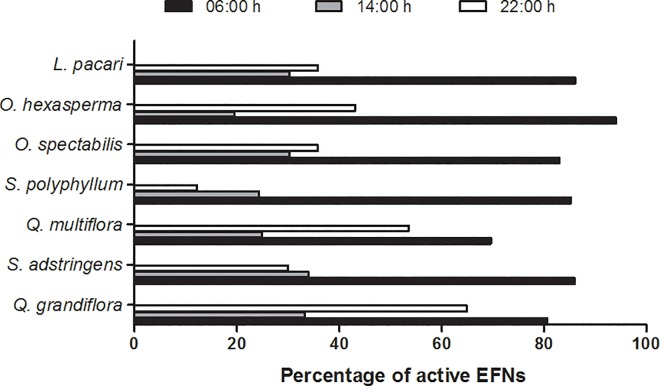
Percentage of active EFNs (n = 210 glands) during all the period of EFNs productivity (since the beginning of their activity until their necrosis) in each of the seven assessed plant species at three distinct times of the day.

We found that some EFNs, even the active ones (because no necrosis was found in their structures), did not produce nectar at least in one time of the day. Observations done at 6:00 am showed the highest active EFN percentage and those performed at 02:00 pm showed the lowest percentage of it (Fig 4). This result was similar in most species (Fig 5).

### Ant foraging

We found 25 ant species foraging on the herein studied group of plants (Table 2). The plant species presenting the greatest ant richness were *S*. *adstringens* and *O*. *spectabilis*, with 10 species each. *Lafoensia pacari* and *Q*. *grandiflora* were the poorest species (five ant species each). *Stryphnodendron polyphyllum* and *O*. *spectabilis* were visited by ants more often, whereas *L*. *pacari* and *Q*. *grandiflora* were less visited by them. However, we found no difference in the relative frequency of ant species depending on the plant species (one-way ANOVA, F_[__6__,_ _13__]_ = 2.37; p = 0.085).

**Table 2:** Relative frequency (%) and number of records of ant species found in the group of plants (seven species) assessed at three distinct times of the day.

Ant Species	*Activity period	No. of records (%)	Plant Species associated
**Formicinae**			
* Brachymyrmex* sp.1	morning, afternoon	5(3.25)	Sp, Os, Oh
* Brachymyrmex* sp.2	night	3(1.95)	Oh, Sp
* Camponotus* sp.1	night	4(2.60)	Sa, Qg, Lp
* Camponotus* sp.2	morning, night	8(5.20)	Sa, Qm, Os, Oh
* Camponotus* sp.3	night	3(1.95)	Sp
* Camponotus* sp.4	night	1(0.65)	Sa
* Camponotus* sp.5	night	1(0.65)	Lp
* C*. *crassus* Mayr, 1862	morning, afternoon, night	33(21.40)	Sa, Qm, Sp, Os
* C*. *blandus* (Smith. F., 1858)	morning, afternoon	8(5.20)	Qm, Sp, Os, Oh
* C*. *trapeziceps* Forel, 1908	afternoon	1(0.65)	Qm
**Myrmicinae**			
* Crematogaster* sp.1	morning, night	5(3.25)	Qm, Sp
* Crematogaster* sp.2	morning, afternoon	2(1.30)	Qg, Sp
* C*. *erecta* Mayr, 1866	morning, afternoon	1(0.65)	Qg
* C*. *bruchi* Forel, 1912	morning, afternoon, night	15(9.75)	Qm, Oh, Lp
* Solenopsis* sp.1	morning, afternoon, night	5(3.25)	Qm, Os
* Solenopsis* sp.2	morning, afternoon, night	10(6.50)	Sa, Qg, Os, Oh
* Cephalotes pusillus* (Klug, 1824)	morning, afternoon	31(20.10)	Sa, Qm, Sp, Os, Oh, Lp
* Pheidole* sp.1	morning	1(0.65)	Sa
**Dolichoderinae**			
* Azteca* sp.1	afternoon, night	2(1.30)	Oh
**Pseudomyrmicinae**			
* Pseudomyrmex gracilis* (Fabricius, 1804)	afternoon	1(0.65)	Sa
* P*. *flavidulus* (Smith. F., 1858)	morning, afternoon	7(4.55)	Sa, Sp, Os, Lp
**Ectatomminae**			
* Ectatomma tuberculatum* (Olivier, 1792)	morning	2(1.30)	Qg, Oh
* E*. *planidens* Borgmeier, 1939	morning, afternoon	2(1.30)	Os
* Gnamptogenys semiferox* Brown, 1958	morning	2(1.30)	Os
**Ponerinae**			
* Neoponera villosa* (Fabricius, 1804)	morning	1(0.65)	Sa

* Morning (06:00 h); afternoon (14:00 h); night (22:00 h). (Qg) *Qualea grandiflora*, (Sa) *Stryphnodendron adstringens*, (Qm) *Q*. *multiflora*, (Sp) *S*. *polyphyllum*, (Os) *Ouratea spectabilis*, (Oh) *O*. *hexasperma* and (Lp) *Lafoensia pacari*.

**Fig 6:**
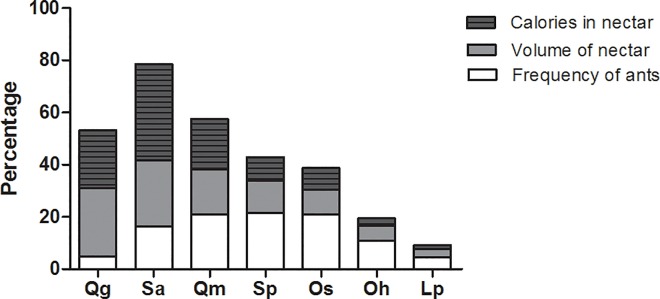
Percentage of relative frequency of ants, total volume of nectar and quantity of calories produced at the EFN in the group of plants (seven species). The letters on the X-axis represent the plant species: (Qg) *Qualea grandiflora*, (Sa) *Stryphnodendron adstringens*, (Qm) *Q*. *multiflora*, (Sp) *S*. *polyphyllum*, (Os) *Ouratea spectabilis*, (Oh) *O*. *hexasperma* and (Lp) *Lafoensia pacari*.

*Camponotus crassus* was the most common (frequent and abundant) ant species on the plants, whereas *Cephalotes pusillus* visited almost all the plant species, except for *Q*. *grandiflora*. There were different ant species foraging on plants each time of the day (Table 2). Some ant species foraged at day light (13 species) and others only at night (five species). *Camponotus crassus*, *Crematogaster bruchi*, *Solenopsis* sp.1 and *Solenopsis* sp.2 were the only species found on the plants all day long (Table 2). We found the highest frequency of ants on plants at 6:00 am (41.7%). There was no significant link between the extrafloral nectar production (volume or quantity of calories) and the frequency of ants (Pearson’ correlation r = 0.06; p = 0.56, for volume; r = 0.15; p = 0.86, for calories; n = 07) (Fig 6).

## Discussion

Our results showed that plant species produce different amounts and sugar concentrations of extrafloral nectar. These results corroborate the initial hypothesis that some plant species may be more attractive to ants than others. According to Blüthgen and Fiedler [36], both nectar quantity and quality may be crucial factors for the foraging activity of these insects on plants. Moreover, according to Ness et al. [37], the presence of extrafloral nectar might make some ant species more aggressive. The extrafloral nectar may significantly increase the adaptive value of ant colonies [28]. Thus, ants compete for plant resources such as nectar and it has driven evolutionary specialization to both actors involved in these interactions. Such competition is the determining factor for the convergence or parallelism of ant-plant interactions [38].

Other studies also found difference in EFN productivity among species [1, 39] depending on the different time of the day [27, 32, 40]. The EFN productivity is determined through plant genotype [41–42], but it may be affected by herbivores due to the induced defense [32, 43–44], and also by abiotic factors such as temperature and moisture [45–47].

The genotype can determine species with larger glands and larger cavities to retain nectar and, therefore, more productive EFNs, as it may be evidenced in the current study. *Qualea grandiflora* and *S*. *adstringens* have larger EFNs than the other species assessed in the present study (see [5]) and they presented the largest nectar production. On the other hand, the EFNs of *L*. *pacari*, *O*. *spectabilis* and *O*. *hexasperma*, (which have smaller nectaries) do not have a cavity to retain nectar. Consequently, these species produced less extrafloral nectar. Thus, the genetic, morphological and phenotypical features of the plants directly affect the final floral and extrafloral nectar production [35, 48–49].

The daily variation in EFN productivity found in the herein studied group of plants may be associated with several factors. Climate conditions such as temperature and moisture may influence both the floral and the extrafloral nectar concentrations [45–47]. According to Nicolson *et al*. [45], high temperatures may increase water evaporation in the nectar, so the nectar gets more concentrated in the warmest times of the day. The temperature may have influenced the extrafloral nectar concentration in the current study, since the nectar from all the species showed the highest concentrations between 02:00 pm and 10:00 pm. In addition, the environmental conditions may have influenced physiological rhythms in plants, for instance: stomata opening and closing to regulate water stress and nutrient absorption from the soil [45]. Some species have stomatal cells in their EFNs [5, 50] and it may directly influence their activity and nectar concentration. Three out of the seven plant species assessed in the current study have stomatal cells in their EFNs: *O*. *spectalibis*, *O*. *hexasperma* and *L*. *pacari* (see [5]). Thus, the EFN productivity of these species may be influenced by the water evaporation from the nectar and by the stomatal closure during higher temperature periods. The nectar becomes so concentrated at these time intervals that the solutes crystallize over the nectaries and it makes the nectar imperceptible to the human eye and impossible to be collected through microcapillaries as we have shown in *O*. *hexasperma* and *L*. *pacari*. On the other hand, stomata opened at times of lower temperature in most species and it increased the water and nutrient flow in the plant and diluted the extrafloral nectar.

Likewise, the difference in the number of active EFNs depending on the time of the day may be explained by the environmental conditions and by the physiological adaptation of the plants. The floral nectar is secreted in specific secretion and absorption rhythms, which are known as dynamic production [35]. According to Nicolson and Nepi [51], this dynamic is often associated with climate conditions or with the behavior of visitors. Like floral nectaries, which synchronize their productivity and the behavior of their pollinators, the EFN productivity may be an evolutionary adaptation of each species to its associates and to habitat, as predicted by optimal defense theory; plant defense production is synchronized with herbivore activity [52–53]. According to these authors, the plant defense production is synchronized with the activity of herbivores. Thus, the extrafloral nectar production may increase in periods of great herbivores activity to attract predatory insects that feed of this nectar. Therefore, these plants are more protected against herbivores under such conditions. In addition, Bentley [54] suggested that the plants control the extrafloral nectar production to minimize their production costs. This model can be similarly shortened why these species and individuals control their anti-herbivory defense production (see [55–56]).

The greatest ant richness was evident during the highest EFN productivity, so, there is greater co-occurrence of species on plants during this time. Lange et al. [2] also showed that the greater extrafloral nectar availability in the environment increases the co-occurrence of ant species on plants. Moreover, despite the importance of the extrafloral nectar to the ants, studies have shown that the frequent resource monopolization by ants is uncommon in plants [57]. The interactions between ants and plants that have EFNs is mostly opportunistic, i.e., different ant species share the same food sources [19, 21, 58].

Besides resource availability, other factors needed to be taken into consideration when we assessed the link between food resource and foraging ants. Studies showed that the patterns of foraging ants that feed on nectar or on honeydew are related to the nutritional demands of these ants [59–60], to the nutritional features of the food resources [61–63], to the spatial distribution of resources [61] and to the nest-to-food distance [62–63]. Furthermore, the ecological success of these ants depends on their ability to adjust their foraging strategies to their resources and environmental constraints [64]. According to Cerdá et al. [65], temperature is a determining factor for ants to choose the time for foraging. Differences in foraging times among species are often associated with the different temperature and humidity ranges tolerated by each species, especially in tropical habitats [66]. These ecophysiological differences among species may reduce the interspecific competition for predictable food sources [64, 67]. Such temporal partition per resource was found in other studies [36, 39, 68]. We found turnover of species in all the periods (morning, afternoon and night).

However, despite the temporal partition found in the present study, some species such as *C*. *crassus* foraged in three different times of the day. The species belonging to this genus prevail on these plants and they are often found in great abundance. These species are generalist foragers that set partnerships with honeydew producers [64, 69]. The dominance of these species in Cerrado EFNs-bearing plants was also observed in other studies [2, 13, 70], and it demonstrates their strong adaptation to such environment [25].

These results suggest that although we found no significant relation between plants presenting greater extrafloral nectar production and the higher frequency of ants, we showed that periods of greater resource availability (abundance of active EFNs and higher volume of nectar and calories) favored the greater richness and higher frequency of ants on plants. These results suggested that the ant-plant system regarding EFNs is a complex process, which presents several peculiarities that need to be understood within the community context. It is also necessary to get to know the natural history of all the species involved in this interaction. We expect that the findings shown in the current study may provide important information to other studies about the ant-plant interactions and about the importance of both groups to the determination of mutualistic interaction structures.
